# Slow progress towards pneumonia control for children in low-and-middle income countries as measured by pneumonia indicators: A systematic review of the literature

**DOI:** 10.7189/jogh.12.10006

**Published:** 2022-10-25

**Authors:** Alicia Quach, Hollie Spence, Cattram Nguyen, Stephen M Graham, Claire von Mollendorf, Kim Mulholland, Fiona M Russell

**Affiliations:** 1 Asia-Pacific Health Group, Murdoch Children’s Research Institute, Victoria, Australia; 2 Department of Paediatrics, The University of Melbourne, Victoria, Australia; 3 Murdoch Children’s Research Institute, Victoria, Australia; 4 The Royal Children’s Hospital, Parkville, Victoria, Australia; 5 Burnet Institute, Melbourne, Victoria, Australia; 6 London School of Hygiene and Tropical Medicine, London, United Kingdom

## Abstract

**Background:**

The integrated Global Action Plan for Prevention and Control of Pneumonia and Diarrhoea (GAPPD) has the goal of ending preventable childhood deaths from pneumonia and diarrhoea by 2025 with targets and indicators to monitor progress. The aim of this systematic review is to summarise how low-and-middle income countries (LMICs) reported pneumonia-specific GAPPD indicators at national and subnational levels and whether GAPPD targets have been achieved.

**Methods:**

We searched MEDLINE, Embase, PubMed and Global Health Databases, and the World Health Organization (WHO) website. Publications/reports between 2015 and 2020 reporting on two or more GAPPD-pneumonia indicators from LMICs were included. Data prior to 2015 were included if available in the same report series. Quality of publications was assessed with the Quality Assessment Tool for Quantitative Studies. A narrative synthesis of the literature was performed to describe which countries and WHO regions were reporting on GAPPD indicators and progress in GAPPD coverage targets.

**Results:**

Our search identified 17 publications/reports meeting inclusion criteria, with six from peer-reviewed publications. Data were available from 139 LMICs between 2010 and 2020, predominantly from Africa. Immunisation coverage rates were the indicators most commonly reported, followed by exclusive breastfeeding rates and pneumonia case management. Most GAPPD indicators were reported at the national level with minimal reporting at the subnational level. Immunisation coverage (*Haemophilus influenzae*, measles, diphtheria-tetanus-pertussis vaccines) in the WHO Europe, Americas and South-East Asia regions were meeting 90% coverage targets, while pneumococcal conjugate vaccine coverage lagged globally. The remaining GAPPD indicators (breastfeeding, pneumonia case management, antiretroviral prophylaxis, household air pollution) were not meeting GAPPD targets in LMICs. There was a strong negative correlation between pneumonia specific GAPPD coverage rates and under-five mortality (Pearson correlation coefficient range = -0.74, -0.79).

**Conclusion:**

There is still substantial progress to be made in LMICs to achieve the 2025 GAPPD targets. Current GAPPD indicators along with country reporting mechanisms should be reviewed with consideration of adding undernutrition and access to oxygen therapy as important indicators which impact pneumonia outcomes. Further research on GAPPD indicators over longer time periods and at subnational levels can help identify high-risk populations for targeted pneumonia interventions.

Global under-five mortality rates (U5MR) have fallen substantially over the past three decades [1,2]. However, a large number of deaths persist with pneumonia causing 14% of U5M in 2019 [3]. The vast majority occurred in low-and-middle income countries (LMICs) with many of these deaths preventable. The health-related goals outlined in the Sustainable Development Goals (SDGs) are centred around achieving equity, between and within nations. To achieve this, substantial efforts are needed to eliminate preventable deaths in the most vulnerable populations.

The Global Action Plan for Prevention and Control of Pneumonia (GAPP) published in 2009, aimed to raise awareness of pneumonia as a major cause of childhood death and scale up interventions to prevent and control the disease [4]. The integrated Global Action Plan for Prevention and Control Pneumonia and Diarrhoea (GAPPD) published in 2013 included diarrhoea as the other major cause of preventable childhood mortality and outlined recommendations to address these diseases together [5]. GAPPD targets were drawn from evidence-based interventions to reduce mortality and focus on protection against diseases, as well as prevention and treatment of pneumonia and diarrhoea.

The pneumonia-related end goals of the GAPPD include a reduction of U5MR caused by pneumonia to less than three per 1000 live births and a 75% reduction in the incidence of severe pneumonia by 2025 from 2010 levels [5]. To reach these goals, the GAPPD set pneumonia specific coverage targets for 2025: 90% full-dose coverage of vaccinations by age 12 months (first dose measles vaccine, three doses of *Haemophilus influenzae* type B (Hib3) vaccine, three doses of pneumococcal conjugate vaccine (PCV3), three doses of diphtheria-tetanus-pertussis (DTP3) vaccine); 90% access to timely pneumonia case management; and at least 50% coverage of exclusive breastfeeding (EBF) during the first six months of life [5]. Other targets included reducing indoor air pollution and virtual elimination of paediatric HIV transmission. The GAPPD report made recommendations on indicators for countries to monitor to inform progress made towards these targets.

This systematic review aims to summarise the reported progress of pneumonia-specific GAPPD indicators at national and subnational levels in LMICs since the introduction of the SDGs in 2015 and to determine whether there is a correlation between pneumonia-specific GAPPD indicator coverage rates and U5MR.

## METHODS

### Search strategy and selection criteria

Using PRISMA reporting guidelines [6], we searched the literature in August 2020 to identify publications/reports that were published in English language reporting on at least two of the pneumonia specific GAPPD indicators from a LMIC, with data from 2015 onwards. MEDLINE (Ovid) and Embase (Ovid) databases were searched using Medical Subject Heading (MeSH) terms and/or keywords. PubMed was searched using keywords only, to retrieve e-publications and items not indexed on MEDLINE. The PubMed search strategy was adapted for use in the Global Health (CAB direct) database and the World Health Organization (WHO) website and library database. Additional peer-reviewed publications were identified through hand-searching of reference lists of key articles. The search strategy used for MEDLINE (Ovid) is available in Appendix S1 in the **Online Supplementary Document**.

Eleven pneumonia-specific GAPPD indicators were included for this review: immunisation coverage rates of measles, Hib, PCV, DTP; EBF rates for the first six months; continued breastfeeding rates at one year of age; rates of adequate complementary feeding from 6-23 months; antiretroviral prophylaxis coverage rates for HIV positive pregnant women; percentages of households using solid fuels for cooking; percentages of children with suspected pneumonia taken to an appropriate health care provider; and percentages of children receiving appropriate antibiotic treatment for suspected pneumonia. Immunisation coverage was considered as a single indicator irrespective of how many vaccines were reported; and EBF at six months and continued breastfeeding at one year was also considered as a single indicator. To be eligible for inclusion, the focus of the publications or reports needed to be on pneumonia outcomes and/or child survival/mortality. All study designs and reports from grey literature were included. These included reviews and monitoring data sets specific to GAPPD indicators. Small scale studies from single sites and studies that reported only on diarrhoea specific GAPPD indicators, not overlapping with pneumonia indicators, were excluded.

The screening process was performed independently by two reviewers (AQ and HS) using Covidence systematic review software [7]. The titles and abstracts were screened and excluded if inclusion/exclusion criteria were not met. Full texts of remaining publications/reports were assessed for eligibility. Conflicts were resolved by discussion of the publication/report by the two reviewers. If consensus was not reached, a third reviewer (FR) was consulted.

### Data analysis

Data were extracted from publications/reports into an Excel spreadsheet format. The main outcomes of interest were the number of GAPPD indicators reported and coverage rates of individual GAPPD indicators. Secondary outcomes extracted included pneumonia mortality rates and U5MR. Quality and bias of original studies included in the review were assessed independently by two reviewers (AQ and HS) using the Effective Public Health Practice Project Quality Assessment Tool for Quantitative Studies [8].

Descriptive summaries were made to describe which countries and WHO regions reported on GAPPD indicators. Mean or median coverage rates, dependent on the distribution, of individual pneumonia specific GAPPD indicators, were calculated with trends shown over the period of 2010 to 2020 where available. Where combined GAPPD-pneumonia scores, defined as the average percentage of the combined coverage rates of the GAPPD-pneumonia indicators, were reported, a Pearson correlation coefficient was calculated, to determine the association between national-level combined GAPPD-pneumonia coverage rates and U5MR, separately by year.

## RESULTS

**Figure 1:**
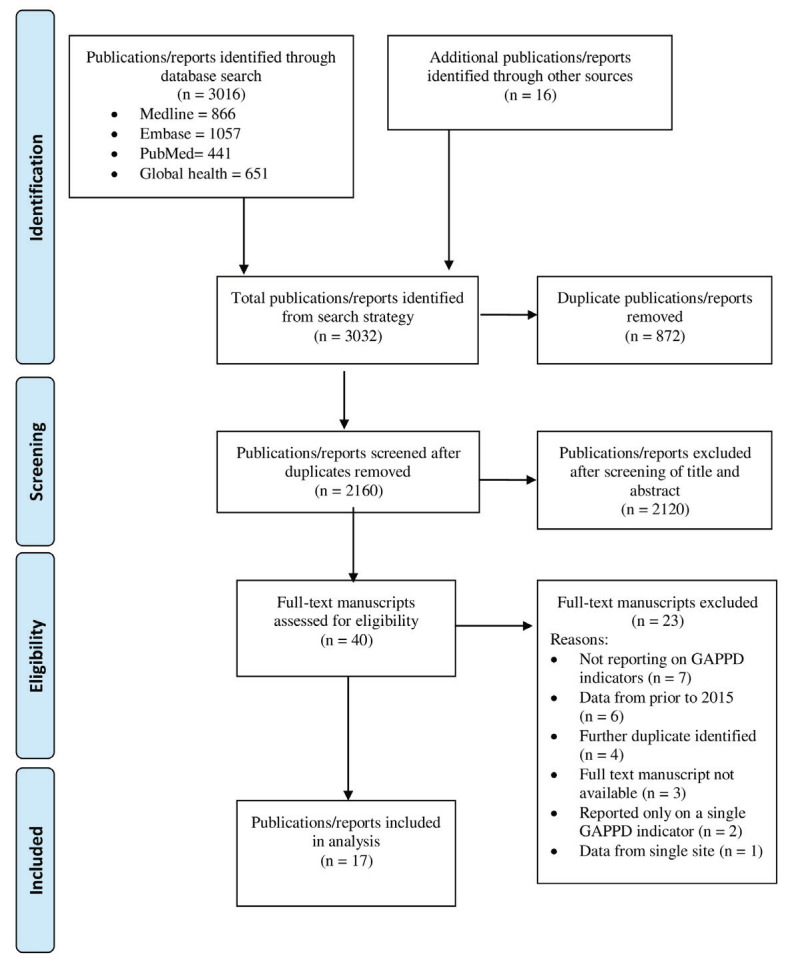
Prisma flow diagram for search strategy of review of progress of GAPPD indicators.

The search strategy identified 3032 publications/reports. Following removal of duplicates, 2160 titles and abstracts were screened, with 2120 excluded. The remaining 40 full-text manuscripts were assessed for eligibility, with 17 publications/reports deemed eligible to be included in the review (**Figure 1**).

The search strategy identified two sources (WHO GAPPD monitoring database [9] and IVAC/JHBSPH Pneumonia and Diarrhoea Progress Reports [10-18]) with data on GAPPD indicators from 2000 to 2016 and 2011 to 2020, respectively. To correspond with the year following release of the original GAPP report in 2009 and to portray more detailed trends over time, the WHO GAPPD monitoring database had data extracted from 2010 to 2016. All IVAC/JHBSPH reports (2011 to 2020) were included in the data extraction, including the 2020 IVAC/JHBSPH report [19], which was released after the search strategy for this systematic review was performed.

### Study description

**Table 1** summarises the characteristics of the included publications/reports. The WHO GAPPD monitoring database draws upon publicly available data sources to periodically update the GAPPD indicator progress of 139 LMICs [9]. The full list of the 139 countries is available in Appendix S2 in the **Online Supplementary Document**. The IVAC/JHBSPH Pneumonia and Diarrhoea Progress Reports are annual reports of the 15 countries with the highest number of under-five pneumonia and diarrhoea deaths. From 2011 to 2020, the majority (21/28) of these countries were from Africa. The remaining publications that met inclusion criteria included original studies and reviews all from Nigeria or Ethiopia. The data presented in the WHO GAPPD monitoring database and IVAC/JHBSPH reports used similar publicly available data sources and reported at the national level. Five out of the six peer-reviewed publications had data at the subnational level.

**Table 1:** Summary of included publications/reports on the progress of Global Action Plan for Prevention and Control of Pneumonia and Diarrhoea (GAPPD) indicators

First author/publication	Year of publication	Study design	Country	Study setting	Data source(s)	Level of data reporting	Age group of participants	Quality assessment score†
WHO GAPPD monitoring data [9]	2017 (most recent update)	N/A	139 LMICs*	N/A	WHO/ UNICEF immunisation coverage estimates, USAID DHS, UNICEF MICS, WHO nutritional landscape information system, UNAIDS, CHERG.	National	<5 y	Weak
IVAC & JHBSPH Pneumonia and diarrhoea progress reports [10-19]	2011–2020 (10 annual reports)	Review	India, Nigeria, Pakistan, Democratic Republic of the Congo, Angola, Ethiopia, Indonesia, Chad, Afghanistan, Niger, China, Sudan, Bangladesh, Somalia, Tanzania, Uganda, Cote d’Ivoire, Mali, Central African Republic, South Sudan, Sierra Leone, Guinea, Benin, Haiti, Cameroon, Mauritania, Kenya, Burkina Faso.	Community based data collection	WHO/ UNICEF immunisation coverage estimates, USAID DHS, UNICEF MICS, UN-IGME	National	<5 y	Weak
Geleta, D [20]	2016	Case-control study	Ethiopia	Health facility	Study questionnaires, health facility records	Subnational (Kersa district)	2-59 mo	Moderate
Oresanya, O [21]	2019	Cross-sectional study	Nigeria	Community based	Study developed household surveys	Subnational (Niger state)	2-59 mo	Weak
Obi, C [22]	2019	Mixed methods: review + qualitative study	Nigeria	Community based	Quantitative: National nutrition and health survey, WHO/ UNICEF national immunisation coverage estimates, National AIDS and STI control program, Nigeria DHS, UN-IGME Qualitative: stakeholders involved in intervention programs for pneumonia and diarrhoea control	National	Quantitative: <5 y, Qualitative: N/A	Weak
Tariku, A [23]	2020	Cross-sectional study	Ethiopia	Community and health facilities	Study questionnaire, child vaccination records	Subnational (four regions)	2-59 mo	Weak
Iuliano, A [24]	2019	Review	Nigeria	Community based	UNICEF MICS, USAID DHS, IVAC, Save the Children – fighting to breathe report, original study reports	National and subnational (Lagos and Jigawa states)	<5 y	Weak
Lema, B [25]	2019	Cross-sectional study	Ethiopia	Community based	Study questionnaire	Subnational (Munesa district)	2-59 mo	Weak

mo – months, y – years

*Full list of included countries available in Appendix S2 in the **Online Supplementary Document**.

†Global rating score rated through the EPHPP Quality Assessment Tool for Quantitative Studies [8].

### Study outcomes

**Table 2** summarises the 11 pneumonia-specific GAPPD indicators collected in the WHO GAPPD monitoring database categorised by WHO region, and the respective total U5M. Data availability was mostly complete for immunisation coverage. The lack of PCV data reflected countries that were yet to introduce it into their routine schedule rather than missing data. Aside from ART prophylaxis, data for the remaining GAPPD indicators were sparse. From 2010 to 2016, no more than 30/139 countries had data entered on breastfeeding, pneumonia case-management or household air pollution in any one year.

**Table 2:** Summary of Global Action Plan for Prevention and Control of Pneumonia and Diarrhoea (GAPPD)-pneumonia indicators reported in WHO GAPPD monitoring data*

Year	WHO region (Number of countries represented)	Countries reporting on pneumonia-specific GAPPD indicator*											Total under-five deaths (n)	Total under-five pneumonia deaths (n)
**Immunisation**				**Breastfeeding**		**Complementary feeding**	**Care seeking**	**Antibiotics**	**ART prophylaxis**	**Household solid fuel use**
**DTP3**	**Hib3**	**Measles**	**PCV3**	**EBF**	**CBF**					
2010	AFR (46)	45 (83.0%)	41 (83.0%)	45 (78.0%)	3 (97.0%)	17 (39%)	17 (92.9%)	2 (5.9%)	17 (52.1%)	16 (41.5%)	42 (29.5%)	17 (97.1%)	3 154 198	547 264
	AMR (26)	26 (94.0%)	25 (94.0%)	26 (94.5%)	8 (58.5%)	4 (45.8%)	4 (41.6%)	1 (59.6%)	5 (65.3%)	3 (69.9%)	21 (56.0%)	3 (13.5%)	233 936	25 778
	EMR (15)	15 (88.0%)	10 (81.5%)	15 (85.0%)	1 (1%)	2 (53.7%)	2 (86.0%)	0†	2 (69.3%)	1 (63.9%)	9 (8.0%)	1 (84.2%)	957 111	172 441
	EUR (21)	21 (94.0%)	17 (91.0%)	21 (97.0%)	2 (81%)	3 (31.8%)	3 (44.2%)	1 (31.7%)	3 (81.2%)	3 (81.6%)	8 (85.0%)	2 (6.2%)	107 351	13 771
	SEAR (11)	11 (91.0%)	2 (96.5%)	11 (88.0%)	0 (0%)	1 (49.0%)	1 (93.0)	0†	1 (74.2%)	1 (48.7%)	5 (19.0%)	1 (39.5%)	2 028 562	343 430
	WPR (20)	20 (90.5%)	19 (89.5%)	20 (89.0%)	3 (70.0%)	2 (69.6%)	2 (82.9%)	0†	2 (75.5%)	2 (55.8%)	6 (21.5%)	2 (78.1%)	413 700	62 847
2011	AFR (46)	46 (82.0%)	43 (83.0%)	46 (80.5%)	12 (46.0%)	11 (26.9%)	11 (85.0%)	7 (5.7%)	11 (41.3%)	11 (30.4%)	42 (45.0%)	16 (86.9%)	3 102 185	538 118
	AMR (26)	26 (91.5%)	25 (91.0%)	26 (94.5%)	11 (71.0%)	6 (31.9%)	6 (58.4%)	1 (54.2%)	7 (77.2%)	6 (59.3%)	21 (65.0%)	6 (17.9%)	189 032	24 021
	EMR (15)	15 (87.0%)	11 (81.0%)	15 (84.0%)	2 (32.0%)	2 (14.1%)	2 (50.4%)	0†	2 (66.9%)	2 (62.3%)	9 (5.0%)	2 (0.8%)	966 127	170 025
	EUR (21)	21 (96.0%)	20 (94.5%)	21 (97.0%)	3 (96.0%)	2 (21.0%)	2 (23.1%)	0†	1 (87%)	1 (76.0%)	8 (80.5%)	2 (51.6%)	104 525	13 199
	SEAR (11)	11 (94.0%)	3 (92.0%)	11 (93.0%)	0 (0%)	2 (67.0%)	2 (93.8)	0†	2 (42.2%)	2 (39.2%)	5 (25.0%)	2 (80.7%)	1 926 336	321 143
	WPR (20)	20 (94.0%)	19 (93.0%)	20 (90.0%)	3 (63.0%)	2 (28.7%)	2 (73.5%)	0†	2 (63.7%)	2 (62.9%)	6 (27.0%)	2 (71.5%)	391 392	58 940
2012	AFR (46)	46 (84.5%)	44 (84.5%)	46 (82.0%)	23 (76.0%)	7 (23.3%)	7 (89.1%)	4 (5.2%)	6 (46.2%)	6 (29.4%)	42 (50.5%)	9 (96.9%)	3 004 435	518 271
	AMR (26)	26 (95.0%)	25 (95.0%)	26 (94.0%)	14 (90.0%)	5 (39.7%)	5 (81.3%)	2 (33.4%)	2 (50.9%)	2 (46.8%)	21 (75.0%)	4 (24.0%)	184 446	22 662
	EMR (15)	15 (81.0%)	12 (81.0%)	15 (83.0%)	5 (73.0%)	3 (22.7%)	3 (62.1%)	1 (33.3%)	2 (70.8%)	2 (64.2%)	9 (6.0%)	2 (31.2%)	949 138	165 963
	EUR (21)	21 (95.0%)	20 (94.5%)	21 (95.0%)	5 (94.0%)	5 (34.3%)	5 (48.4%)	1 (19.6%)	5 (79.2%)	5 (76.7%)	8 (86.0%)	6 (14.6%)	101 493	12 423
	SEAR (11)	11 (94.0%)	5 (94.0%)	11 (88.0%)	0 (0%)	2 (26.9%)	2 (77.2%)	1 (36.6%)	3 (75.3%)	3 (45.4%)	5 (33.0%)	3 (38.1%)	1 808 154	298 750
	WPR (20)	20 (91.0%)	19 (86.0%)	20 (92.5%)	5 (61.0%)	1 (52.2%)	1 (49.7%)	0†	0†	0†	6 (31.5%)	0†	372 170	55 121
2013	AFR (46)	46 (86.0%)	44 (87.5%)	46 (81.5%)	23 (76.0%)	9 (48.5%)	9 (92.0%)	9 (10.2%)	8 (59.2%)	8 (46.9%)	42 (59.0%)	11 (97.1%)	2 959 374	494 792
	AMR (26)	26 (93.5%)	26 (93.5%)	26 (93.5%)	14 (90.0%)	3 (21.5%)	3 (43.7%)	0†	3 (66.0%)	2 (49.1%)	21 (67.0%)	1 (9.3%)	180 938	21 511
	EMR (15)	15 (82.0%)	13 (81.0%)	15 (80.0%)	5 (73.0%)	2 (10.3%)	2 (71.2%)	2 (15.1%)	1 (34%)	1 (53.4%)	9 (7.0%)	1 (35.0%)	934 381	151 735
	EUR (21)	21 (96.0%)	20 (95.5%)	21 (97.0%)	5 (94.0%)	1 (24.5%)	1 (68.2%)	2 (44.1%)	0†	0†	8 (85.5%)	0†	98 214	12 308
	SEAR (11)	11 (93.0%)	10 (87.0%)	11 (91.0%)	0 (0%)	0†	0†	2 (14.3%)	0†	0†	5 (40.0%)	0†	1 709 946	277 804
	WPR (20)	20 (88.0%)	19 (87.0%)	20 (92.0%)	5 (61.0%)	1 (47.1%)	1 (70.4%)	1 (35.3%)	2 (67.2%)	2 (56.7%)	6 (34.5%)	3 (55.5%)	354 033	50 411
2014	AFR (46)	46 (87.0%)	45 (87.0%)	46 (81.5%)	32 (76.5%)	12 (52.4%)	12 (89.2%)	8 (13.1%)	13 (58%)	13 (34.3%)	42 (64.0%)	13 (74.6%)	2 878 147	470 377
	AMR (26)	26 (92.5%)	26 (92.5%)	26 (94.0%)	16 (87.0%)	6 (41.6%)	6 (55.6%)	4 (48.6%)	6 (76.7%)	6 (43.9%)	21 (74.0%)	4 (16.6%)	178 083	21 395
	EMR (15)	15 (81.0%)	14 (79.5%)	15 (79.0%)	7 (73.0%)	2 (47.6%)	2 (84.7%)	2 (19.2%)	2 (58.0%)	2 (60.9%)	9 (7.0%)	1 (58.2%)	897 482	142 915
	EUR (21)	21 (95.0%)	20 (94.0%)	21 (94.0%)	6 (78.0%)	2 (27.0%)	3 (42.7%)	2 (53.6%)	1 (59.7%)	1 (84.7%)	8 (90.5%)	2 (31.8%)	94 186	11 670
	SEAR (11)	11 (93.0%)	10 (92.5%)	11 (94.0%)	0 (0%)	2 (56.1%)	2 (94.8%)	2 (27.4%)	2 (46.1%)	2 (41.7%)	5 (49.0%)	2 (78.5%)	1 635 304	258 577
	WPR (20)	20 (88.0%)	19 (88.0%)	20 (90.5%)	7 (64.0%)	2 (44.8%)	2 (72.8%)	2 (44.7%)	2 (75.0%)	2 (84.8%)	6 (46.0%)	1 (83.9%)	340 655	47 305
2015	AFR (46)	46 (86.5%)	46 (86.5%)	46 (80.5%)	37 (80.0%)	7 (41.4%)	7 (91.6%)	5 (8.7%)	6 (50.0%)	6 (36.1%)	42 (75.5%)	8 (69.5%)	2 819 929	463 096
	AMR (26)	26 (92.0%)	26 (92.0%)	26 (94.0%)	17 (92.0%)	2 (32%)	2 (48.7%)	1 (53.2%)	1 (73.1%)	1 (73.9%)	21 (73.0%)	2 (13%)	174 875	21 275
	EMR (15)	15 (84.0%)	15 (84.0%)	15 (79.0%)	7 (72.0%)	1 (43.3%)	1 (78.4%)	1 (15.5%)	1 (61.6%)	1 (54.4%)	9 (8.0%)	1 (66.7%)	872 020	139 162
	EUR (21)	21 (95.0%)	20 (94.0%)	21 (97.0%)	8 (82.0%)	3 (44.5%)	3 (59.8%)	3 (45.1%)	1 (91.7%)	1 (19.6%)	8 (88.0%)	3 (1.5%)	89 777	11 126
	SEAR (11)	11 (96.0%)	10 (93.5%)	11 (94.0%)	2 (26.5%)	2 (37.2%)	2 (60.6%)	2 (35.8%)	2 (68.9%)	2 (56.7%)	5 (48.0%)	2 (48.8%)	1 558 327	234 841
	WPR (20)	20 (90.0%)	19 (89.0%)	20 (90.0%)	10 (71.0%)	0†	0†	0†	0†	0†	6 (50.0%)	0†	322 066	45 312
2016	AFR (46)	46 (85.0%)	46 (85.0%)	46 (81.5%)	39 (81.0%)	5 (57.5%)	5 (91.8%)	2 (7%)	5 (62.5%)	2 (27.6%)	42 (78.0%)	5 (93.0%)	2 720 055	NA
	AMR (26)	26 (94.0%)	26 (94.0%)	26 (95.0%)	17 (90.0%)	0†	0†	0†	0†	0†	21 (71.0%)	0†	184 373	NA
	EMR (15)	15 (84.0%)	15 (84.0%)	15 (79.0%)	7 (82.0%)	0†	0†	0†	0†	0†	9 (8.0%)	0†	866 120	NA
	EUR (21)	21 (94.0%)	20 (92.5%)	21 (96.6%)	10 (95.5%)	0†	0†	0†	0†	0†	8 (86.0%)	0†	87 844	NA
	SEAR (11)	11 (96.0%)	10 (93.0%)	11 (94.0%)	3 (46.0%)	1 (66.1%)	1 (98.1%)	0†	1 (84.9%)	0†	5 (64.0%)	0†	1 406 679	NA
	WPR (20)	20 (90.5%)	19 (90.0%)	20 (87.0%)	10 (78.5%)	0†	0†	0†	0†	0†	6 (58.0%)	0†	306 708	NA

DTP3 – 3 doses of diphtheria, tetanus, pertussis vaccine, Hib3 – 3 doses of *Haemophilus influenzae* vaccine, PCV3 – 3 doses of pneumococcal conjugate vaccine, ART – antiretroviral therapy, EBF – exclusive breast feeding, CBF – continued breast feeding, NA – data not available

WHO Regions: AFR – African region, AMR – American region, EMR – Eastern Mediterranean region, EUR – European region, SEAR – South-East Asian region, WPR – Western Pacific region.

*Data are reported as: number of countries with data (median coverage of GAPPD indicator).

†No data recorded for the associated GAPPD indicator.

The IVAC/JHBSPH annual reports included seven pneumonia indicators (EBF rates for first six months, coverage rates of measles, DTP3, Hib3, PCV3, care by an appropriate health care provider and appropriate antibiotic treatment for suspected pneumonia). The reports calculated a GAPPD-pneumonia score for each included country, defined as the average percentage of the combined coverage rates of the seven indicators. **Table 3** presents the GAPPD-pneumonia scores between 2011 and 2020 for each included country and their respective number of U5 deaths for that year. Chad and Somalia who persistently had the two lowest GAPPD scores between 2014 and 2020, were also the two countries with the highest U5MR in the same period.

**Table 3:** Summary of Global Action Plan for Prevention and Control of Pneumonia and Diarrhoea (GAPPD)-pneumonia indicators and under-five mortality data reported in IVAC/JHBSPH reports*

Country	WHO region	GAPPD-pneumonia score (%)*										Total number of under-five pneumonia and diarrhoea deaths									
**Total number of under-five pneumonia deaths**									
**2011**	**2012**	**2013**	**2014**	**2015**	**2016**	**2017**	**2018**	**2019**	**2020**	**2011**	**2012**	**2013**	**2014**	**2015**	**2016**	**2017**	**2018**	**2019**	**2020**
Angola	AFR	57	54	58	59	77	60	60	49	54	50			48 000	49 000	54 548	54 429	54 429	29 007	25 609	25 609
												33 078	20 900			17 400	17 000		16 983	16 683	16 683
Benin	AFR	-	-	-	-	-	-	-	-	57	-	-	-	-	-	-	-	-	-	9130	-
												-	-	-	-	-	-	-	-		-
Burkina Faso	AFR	49	45	56	-	-	-	-	-	-	-			18 000	-	-	-	-	-	-	-
												24 374	21 800		-	-	-	-	-	-	-
Cameroon	AFR	-	-	-	-	-	-	-	-	64	-	-	-	-	-		-	-	-	17 268	-
												-	-	-	-	-	-	-	-	10 614	-
Central African Republic	AFR	-	-	-	-	-	-	-	-	41	-	-	-	-	-	-	-	-	-	5013	-
												-	-	-	-	-	-	-	-	3129	-
Chad	AFR	-	-	-	31	30	33	29	25	25	28	-	-	-	24 000	30 641	30 635	30 635	29 387	27 496	27 496
												-	-	-		18 800	18 700		18 724	17 981	17 981
Democratic Republic of Congo (DRC)	AFR	45	55	52	52	61	63	63	64	65	50			121 000	83 000	78 422	78 273	78 273	82 017	64 170	64 170
												112 655	87 000			49 100			49 115	38 633	38 633
Cote d’Ivoire	AFR	-	-	-	-	-	-	-	63	59	61	-	-	-	-	-	-	-	20 702	18 651	18 651
												-	-	-	-	-	-	-	13 336	11 074	11 074
Ethiopia	AFR	47	33	41	51	55	60	56	53	52	51			62 000	53 000	46 888	56 962	46 962	45 627	44 692	44 692
												48 892	57 800			32 200	30 700		30 733	30 750	30 750
Guinea	AFR	-	-	-	-	-	-	-	-	42	-	-	-	-	-	-	-	-	-	10 441	-
												-	-	-	-	-	-	-	-	7075	-
Kenya	AFR	57	69	68	66	-	-	-	-	-	-			28 000	29 000	-	-	-	-	-	-
												30 406	20 500			-	-	-	-	-	-
Mali	AFR	-	55	59	-	-	-	-	-	51	61	-		26 000	-	-	-	-	-	21 353	21 353
												-	24 500		-	-	-	-	-	13 426	13 426
Mauritania	AFR	-	-	-	-	-	-	-	-	58	-	-	-	-	-	-	-	-	-	2838	-
												-	-	-	-	-	-	-	-	1789	-
Niger	AFR	45	57	50	48	44	52	47	59	58	59			29 000	25 000	29 164	28 163	28 163	24 405	20 048	20 048
												26 319	21 700			16 100	16 400		16 499	12 828	12 828
Nigeria	AFR	32	28	25	37	39	38	38	33	52	50			23 1000	19 7000	21 0557	21 0219	21 0219	215 306	208 439	208 439
												17 7212	14 3600			16 8300	14 0520		140 520	134 007	134 007
Sierra Leone	AFR	-	-	-	-	-	-	-	-	71	-	-	-	-	-	-	-	-	-	6234	-
												-	-	-	-	-	-	-	-	3746	-
Somalia	AFR	-	-	-	-	26	26	27	27	27	26	-	-	-	-	23 426	23 428	23 428	28 162	25 158	25 158
												-	-	-	-	18 000	17 900		17 937	16 333	16 333
South Sudan	AFR	-	-	-	-	-	-	-	-	39	-	-	-	-	-	-	-	-	-	12 207	-
												-	-	-	-	-	-	-	-	8509	-
Sudan	AFR	-	-	-	66	74	76	75	-	-	-	-	-	-	28 000	24 903	25 087	25 087	-	-	-
												-	-	-		12 600	12 300		-	-	-
Tanzania	AFR	62	-	67	-	84	87	76	78	81	75		-	21 000	-	22 394	22 322	22 322	27 065	25 367	25 367
												25 005	-		-	18 000	17 600		17 624	16 311	16 311
Uganda	AFR	51	60	61	61	-	-	-	75	-	-			25 000	24 000	-	-	-	21 575	-	-
												25 751	24 000			-	-	-	14 578	-	-
Afghanistan	EMR	49	49	56	57	63	66	58	-	-	-			33 000	33 000	30 419	30 394	30 394		-	-
												80 694	48 400			13 400	12 800		-	-	-
Pakistan	EMR	60	57	57	61	60	60	60	63	69	68			111 000	10 9000	10 3760	103 444	103 444	99 644	90 398	90 398
												84 210	79 800			64 100	62 700		62 782	57 625	57 625
Haiti	AMR	-	-	-	-	-	-	-	-	46	-	-	-	-	-	-	-	-	-	5614	-
												-	-	-	-	-	-	-	-	3889	-
India	SEAR	39	39	39	42	45	53	66	65	67	71			436 000	31 8000	297 114	296 279	296 279	260 990	23 3240	233 240
												371 605	39 6700			168 300	158 000		158 176	141 970	141 970
Indonesia	SEAR	38	42	40	47	47	55	56	55	54	63			29 000	30 000	33 737	33 551	33 551	27 582	27 422	27 422
												38 331	21 900			21 400	20 000		20 084	19 672	19 672
Bangladesh	SEAR	45	-	-	65	59	65	74	74	74	81		-	-	26 000	24 571	24 541	24 541	24 022	21 166	21 166
												25 978	-	-		18 300	16 900		16 960	13 656	13 656
China	WPR	45	56	56	57	56	45	56	54	54	55			50 000	41 000	27 114	27 113	27 113	25 830	24 252	24 252
												62 229	54 700			22 700	20 800		20 894	19 555	19 555

WHO Regions: AFR – African region, AMR – American region, EMR – Eastern Mediterranean region, EUR – European region, SEAR – South-East Asian region, WPR – Western Pacific region

*GAPPD-pneumonia score – calculated average of coverage rates of seven pneumonia specific indicators (exclusive breastfeeding <6 months, measles coverage, DTP3 coverage, Hib3 coverage, PCV3 coverage, care seeking and antibiotic treatment for pneumonia). (-) – Country was not included in the corresponding year’s report. Empty cells – data not reported

**Table 4** summarises the GAPPD indicators reported in the six peer-reviewed publications. The two review articles by Obi et al. [22] and Iuliano et al. [24] reported on almost all GAPPD-pneumonia indicators in Nigeria. The other Nigerian study by Oresanya et al. reported on pneumonia case management in hard-to-reach populations in Niger state [21]. The two Ethiopian studies included a rural health facility case-control study reporting on immunisation, EBF rates and household air pollution [20] and a community level cross-sectional study reporting on immunisation rates and pneumonia case management [23].

**Table 4:** Summary of Global Action Plan for Prevention and Control of Pneumonia and Diarrhoea (GAPPD)-pneumonia indicators reported from original studies*

Author	Study setting	Year of data collection	Number of participants	Study population	Pneumonia-specific GAPPD indicator							Pneumonia rates/mortality	Under-five mortality
**Immunisation**	**Breast-feeding**	**Complementary feeding**	**Care seeking**	**Antibiotics**	**ART prophylaxis**	**Household air pollution**
Geleta, D.	Ethiopia:seven health centres in Kersa district -mainly rural population	2015	378 (pneumonia cases = 189, control = 189)	Children age 2-59 mo attending “sick baby clinics”	DTP3 = 84.7%, Measles = 55.6%, PCV3 = 83.9%, Hib3 = 84.7%	EBF = 47.1%	-	-	-	-	Cooking in living room or next to living room = 44.2%		-
Oresanya, O.	Nigeria: household surveys in six local government areas in Niger state – mainly rural, hard to reach population	2014	899	“Sick” children age 2-59 mo	-	-	-	76%	28.6%	-	-	Pneumonia prevalence = 43.2%	-
		2017	630	“Sick” children age 2-59 mo	-	-	-	89%	60.5%	-	-	Pneumonia prevalence = 51.2%	-
Obi, C.	Nigeria: review of national data	2013 - 2015	N/A	All children <5 y	DTP3 = 49%, Measles = 51%, PCV3 = 13%, Hib3 = 49%	EBF = 25%	18%	35%	49%	30%	-	Pneumonia deaths = 133 000 (2015)	-
Tariku, A.	Ethiopia: household surveys in four regions -Amhara, Oromia, Tigray, SNNPR regions (80% of population)	2016 - 2017	3110	All children age 2-59 mo	DTP3 = 41%, Measles = 41%, PCV3 = 28%, Hib3 = 41%	-	-	46%	27%	-	-	Pneumonia prevalence = 0.7%	-
Iuliano, A.	Nigeria: review of data from two regions - Jigawa (poor, rural population) and Lagos (rich, urban population) states	2010 - 2019	N/A	All children <5 y	2018	2016/2017	2016/2017	2016/2017	2016/2017		2016/2017	2016/2017	2016/2017
					National: DTP3 = 57.2%, PCV3 = 37.5%, Hib3 = 57.2%	National: EBF = 27.2%	National: 16.5%	National: 84.3%	National: 35.5%	-	National: Solid fuel for cooking = 80.6%	National: Under-five ARI mortality = 19.4%, Post-neonatal ARI mortality = 16.6%	National: 120 per 1000 live births
					Jigawa: DTP3 = 38%, PCV3 = 7.9%, Hib3 = 38%	Jigawa: EBF = 34.4%	Jigawa: 5.1% - 13.9%	Jigawa: 84.1%	Jigawa: 67.2%		Jigawa: Solid fuel for cooking = 98.7%	Jigawa: Postneonatal ARI mortality = 16.3 per 1000 live births	Jigawa: 192 per 1000 live births
					Lagos: DTP3 = 92.6%, PCV3 = 54.3%, Hib3 = 92.6%	Lagos: EBF = 63%	Lagos: 18.3%	Lagos: 95.5%	Lagos: 64%		Lagos: Solid fuel for cooking = 3.3%	Lagos: Post-neonatal ARI mortality = 9.2 per 1000 live births	Lagos: 50 per 1000 live births
Lema, B.	Ethiopia: household surveys across 37 kebeles in Munesa district – mainly rural population	2018	344	All children 2-59 mo	Not published	EBF = 78.4%, CBF = 64.4%	-	-	-	-	Fuel for cooking = charcoal (17.5%), wood (74.7), animal dangling (7.8%) Kitchen separate from main house = 71.8%	Pneumonia prevalence = 17.7%	-

DTP3 – 3 doses of diphtheria, tetanus, pertussis vaccine, Hib3 – 3 doses of *Haemophilus influenzae* vaccine, PCV3 – 3 doses of pneumococcal conjugate vaccine, ART – antiretroviral therapy, EBF – exclusive breast feeding, CBF – continued breast feeding, ARI – acute respiratory infection, y – year, mo – months

*(-) – Data not collected for that GAPPD indicator.

**Figure 2:**
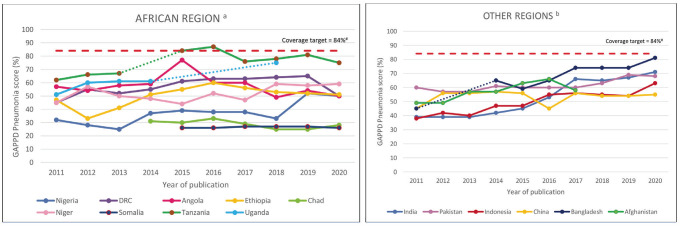
Trends in GAPPD-pneumonia scores, 2011 to 2020 (results from IVAC/JHBSPH Pneumonia and diarrhoea progress reports). GAPPD-pneumonia score – calculated mean percentage of coverage rates of seven pneumonia specific indicators (exclusive breastfeeding <6 months, measles coverage, DTP3 coverage, Hib3 coverage, PCV3 coverage, % of children with suspected pneumonia receiving care by a health care provider and % of children with suspected pneumonia receiving antibiotic treatment). ^#^Coverage target – 2025 pneumonia specific coverage target from the integrated GAPPD report. Calculated by averaging percentage of combined coverage targets of the seven pneumonia indicators. (—) dotted lines – extrapolated trend line for years with no GAPPD scores available. ^a^ Trends of GAPPD scores from African countries are variable, most with minimal progress. ^b^ Countries from other regions showing general upward trend of GAPPD scores, but not yet reaching coverage target.

**Figure 2** shows the trends of the GAPPD-pneumonia scores of the countries included in the IVAC/JHBSPH reports that had five or more data points between 2011 and 2020. A GAPPD-pneumonia coverage target was set at 84% by averaging the 2025 pneumonia specific coverage targets from the integrated GAPPD report [5]. Non-African countries had a general upward trend in GAPPD-pneumonia scores between 2011 and 2020. GAPPD-pneumonia scores in African countries were more variable, with Tanzania being the only country to have met the GAPPD-pneumonia coverage target of 84% in 2015 and 2016. Between 2015 and 2020, Angola showed a steady decline in GAPPD pneumonia scores (from 77% to 50%), whilst Somalia consistently ranked amongst the lowest with no improvement in coverage rates (26%-27%).

**Figure 3:**
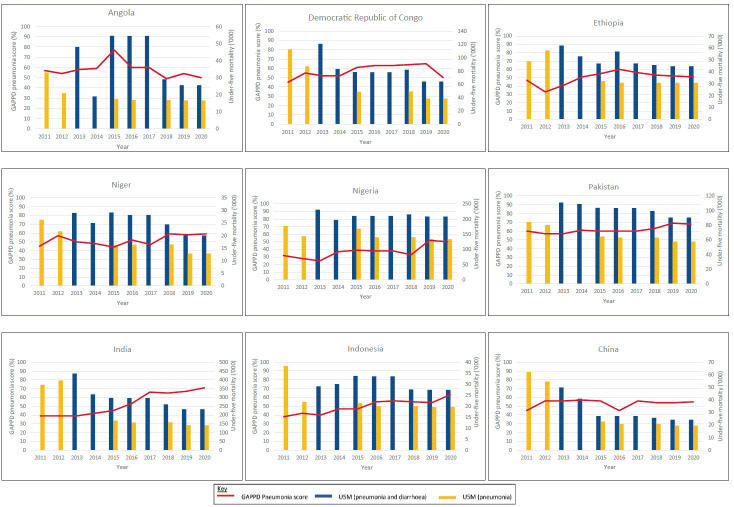
Trends in GAPPD-pneumonia scores and under-five mortality between 2011 and 2020 (results from IVAC/JHBSPH Pneumonia and diarrhoea progress reports). GAPPD-pneumonia score = calculated mean percentage of coverage rates of seven pneumonia specific indicators (exclusive breastfeeding <6 months, measles coverage, DTP3 coverage, Hib3 coverage, PCV3 coverage, % of children with suspected pneumonia receiving care by a health care provider and % of children with suspected pneumonia receiving antibiotic treatment), U5M = under-five mortality. The total number of under deaths due to pneumonia and diarrhoea accordingly. The nine countries displayed were in the top 15 countries with highest burdens of pneumonia and diarrhoeal deaths every year between 2011 and 2020. All countries except Angola showed improvement in GAPPD score. All countries showed downward trend of pneumonia and diarrhoeal deaths.

**Figure 4:**
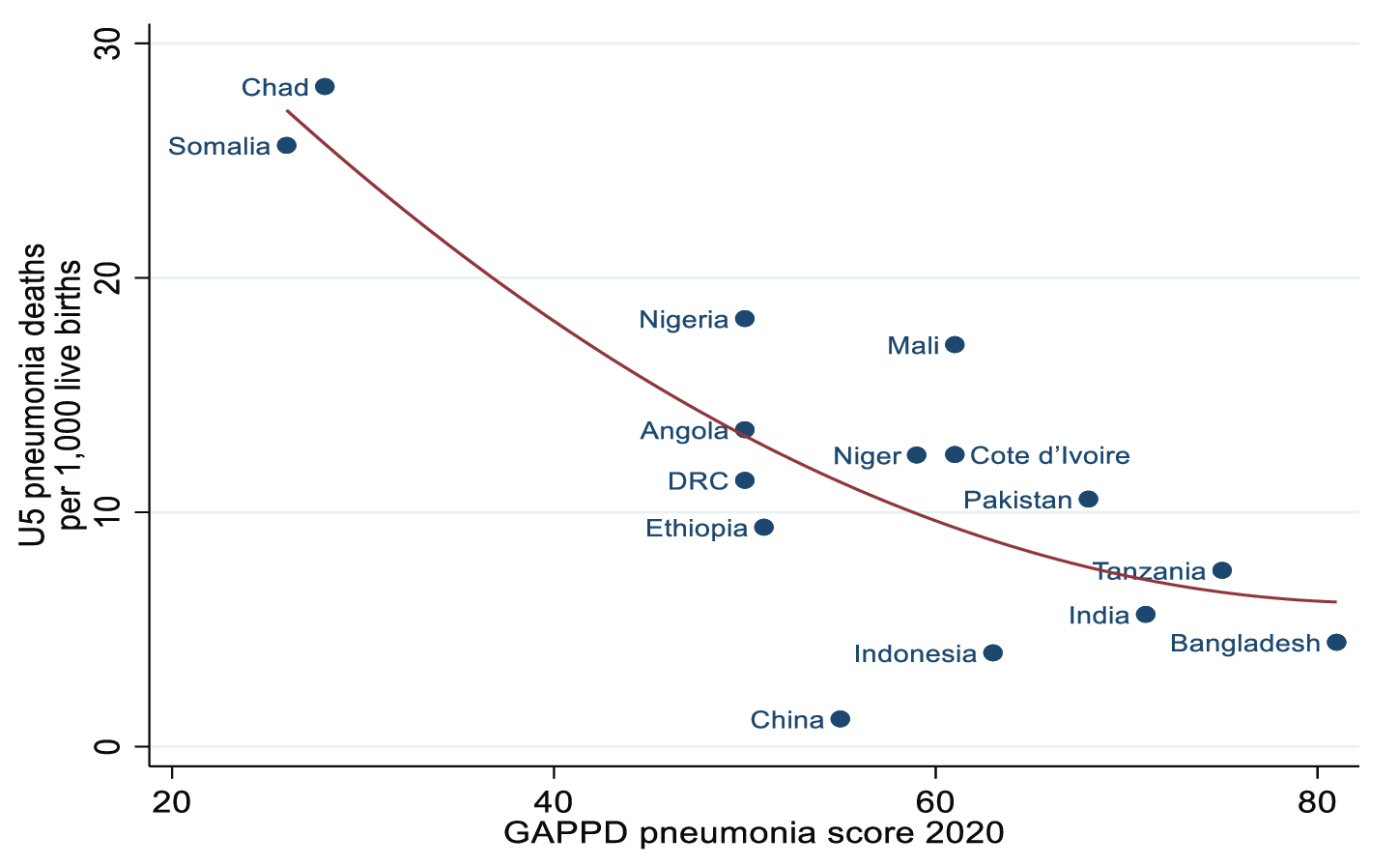
Scatterplot of under-five pneumonia mortality rates against GAPPD-pneumonia scores (results from 2020 IVAC/JHBSPH Pneumonia and diarrhoea progress report). *Mortality data from 2017. Sourced from WHO and Maternal Child Epidemiology Estimation (MCEE).

**Figure 3** shows the trends of the GAPPD-pneumonia scores and U5M for nine countries that were consistently in the top 15 countries with the highest burdens of pneumonia and diarrhoeal deaths between 2011 and 2020. The total number of pneumonia deaths trended downwards in all nine countries over the ten-year period. **Table 5** summarises the percentage change in GAPPD-pneumonia scores and U5M due to pneumonia between 2011 and 2020 for the same nine countries. GAPPD-pneumonia scores were variable with most showing modest increases in coverage rates (change in GAPPD-pneumonia score between 2011 to 2020: median = +10%; range = -7%, +32%). Separate pneumonia and diarrhoea U5MR data were available in 2018 to 2020 IVAC/JHBSPH reports. The Pearson correlation coefficient of GAPPD-pneumonia scores and pneumonia-U5MR for this period ranged from -0.74 to -0.79, suggestive of a strong association between higher GAPPD-pneumonia coverage rates and lower pneumonia-U5MR. **Figure 4** displays the association between GAPPD-pneumonia scores and U5MR due to pneumonia in 2020.

**Table 5:** Summary of percentage change in Global Action Plan for Prevention and Control of Pneumonia and Diarrhoea (GAPPD)-pneumonia scores and under-five mortality (U5M) between 2011 and 2020 (results from IVAC/JHBSPH Pneumonia and diarrhoea progress reports)

Country	% Difference in GAPPD pneumonia score (2011-2020)	% Difference in U5M (pneumonia) (2011-2020)
Angola	-12.3%	-49.6%
Democratic Republic of Congo	+11.1%	-65.7%
Ethiopia	+8.5%	-37.1%
Niger	+31.1%	-51.3%
Nigeria	+56.3%	-24.4%
Pakistan	+13.3%	-31.6%
India	+82.1%	-61.8%
Indonesia	+65.8%	-48.7%
China	+22.2%	-68.6%

**Figure 5:**
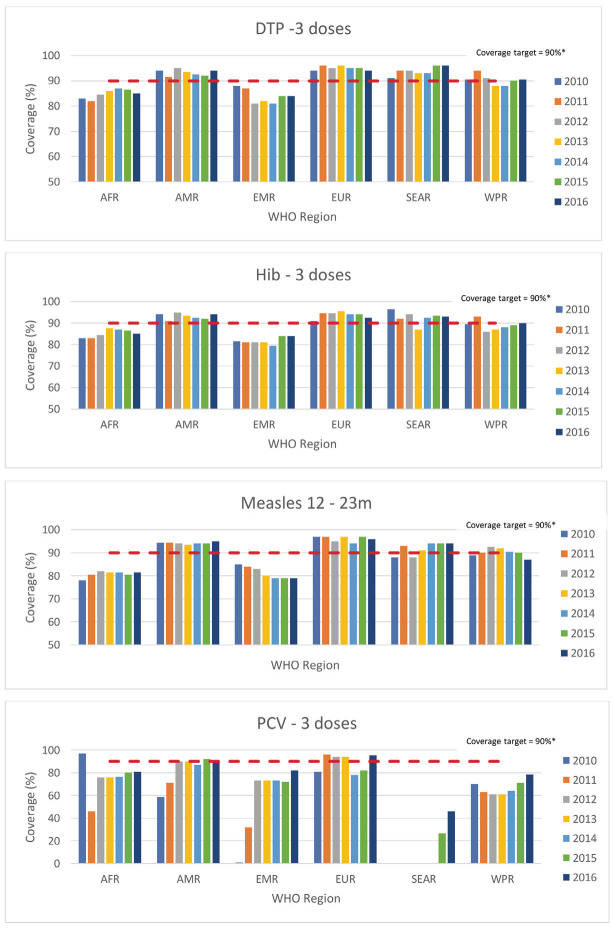
Immunisation median coverage rates by antigen for WHO regions as reported in WHO GAPPD monitoring database. WHO Regions: AFR – African region, AMR – American region, EMR – Eastern Mediterranean region, EUR – European region, SEAR – South-East Asian region, WPR – Western Pacific region. DTP – diphtheria, tetanus, pertussis vaccine, Hib – *Haemophilus influenzae* vaccine, PCV – pneumococcal conjugate vaccine. *Coverage target – 2025 coverage target for immunisations from the integrated GAPPD report.

Immunisation coverage rates were the most widely reported GAPPD indicators. **Figure 5** shows the immunisation coverage rates for the WHO regions between 2010 and 2016 as reported in the WHO GAPPD monitoring database. The European (EUR), Americas (AMR) and South-East Asia (SEAR) regions have met immunisation coverage rate targets of >90% for DTP3, measles and Hib3, with the other WHO regions not far behind (range = 78%-90%). PCV3 immunisation rates are not as uniform. In 2016, 86/139 countries had data available with median coverage rates ranging from 46% (SEAR) to 95.5% (EUR). In the 2020 IVAC/JHBSPH publication, the median immunisation (DTP3, measles, Hib3, PCV3) coverage rates for the 15 countries included in the report were 77%, 76%, 75% and 58%, respectively [19]. The peer-reviewed original studies from Nigeria showed lower coverage rates for PCV3 (13%-37.5%), compared to DTP3 and Hib3 (49%-57.2%) [22,24], noting that PCV-10 was introduced as part of Nigeria’s routine immunisation schedule in December 2014 [26]. Subnational level data showed much higher immunisation coverage rates in Lagos than in Jigawa [24].

Most countries included in the IVAC/JHBSPH publications did not meet the EBF coverage target of 50%. In the 2020 report, only 5/15 countries met this target (range = 0%-65%) [19]. The WHO GAPPD monitoring database had minimal data entered on breastfeeding, with data available for only 6/139 countries in 2016. The median EBF rate was 44.1% (range = 2.8%-84.9%) in 2010 and 61.5% (range = 31.6%-83.1%) in 2016. The median continued breastfeeding rate at one year was 87.3% (range = 18.4%-97.1%) in 2010 and 93.4% (range = 51.4%-98.1%) in 2016 [9]. The EBF rates in Lagos, Nigeria was almost double that of Jigawa, Nigeria [24]. The two Ethiopian studies with EBF data showed higher rates in Munesa district (78.4%) [25] than in Kersa district (47.1%) [20]. Adequate complementary feeding was not well reported across any of the publications/reports.

The rates of care by an appropriate health care provider for suspected pneumonia were highly variable amongst countries and WHO regions, with only a few countries achieving the 90% coverage target (Argentina, Cuba, Ukraine, Armenia and Indonesia) as reported in the WHO GAPPD monitoring database. No country achieved the 90% coverage target for appropriate antibiotic treatment. The Nigerian study in Niger state by Oresanya et al. showed an increase in both care-seeking (76%-89%) and antibiotic treatment (28.6%-60.5%) during the study period of 2014 to 2017 [21]. A rise in health care seeking behaviour in Nigeria was also seen in the IVAC/JHBSPH reports between 2011 to 2020 (45%-75%) [11,19].

**Figure 6:**
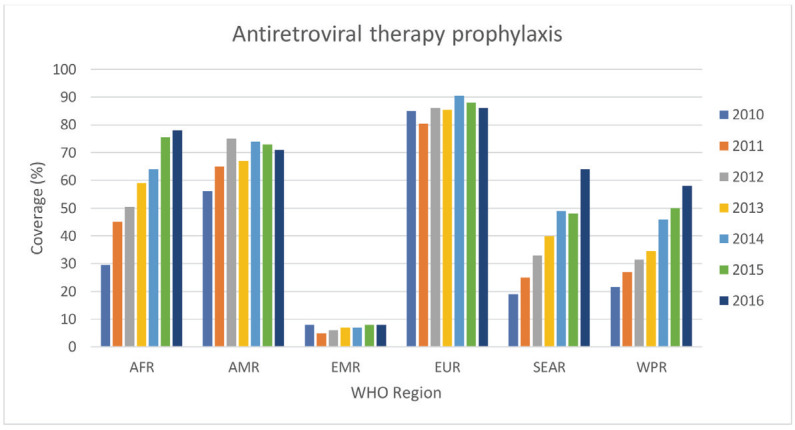
Antiretroviral therapy prophylaxis median annual coverage for WHO regions as reported in WHO GAPPD monitoring database. WHO Regions: AFR – African region, AMR – American region, EMR – Eastern Mediterranean region, EUR – European region, SEAR – South-East Asian region, WPR – Western Pacific region.

The WHO GAPPD monitoring database (91/139 countries) [9] and the Nigerian review by Obi et al [22], were the only publications that reported on ART prophylaxis for HIV-positive pregnant women. The countries and WHO regions with data available are far from universal ART prophylaxis with coverage rates lowest in the Eastern Mediterranean Region (EMR) (8% in 2016, range 4%-51%) [9]. **Figure 6** shows the median coverage rates in the WHO regions from 2010 to 2016. There were no studies reporting on subnational coverage data or hard to reach populations.

Data on household air pollution, reported as the percentage of households using solid fuels for cooking, were sparsely available from the WHO GAPPD monitoring database. Solid fuels were widely used in African (69.5%-97.1%) countries, but less commonly in the Americas (9.3%-24.0%) and Europe (1.5%-51.6%). Three of the peer-reviewed publications reported on solid fuel use, all from Africa, also reflected wide use of solid fuels for cooking at the national level (74.7%-80.6%) [20,24,25]. In Nigeria, rural Jigawa used almost entirely solid fuels (98.7%) compared to their urban counterpart, Lagos (3.3%) [24].

## DISCUSSION

Our review found that many GAPPD indicators are not routinely reported in the published literature. Immunisation coverage was most commonly reported across our included publications/reports, with relatively sparse data for the other GAPPD indicators. National level data using existing large-scale databases were primarily used where reporting occurred. Only two countries (Nigeria and Ethiopia) published data at the subnational level. Most GAPPD coverage targets are not being reached in many LMICs with the exception of 90% immunisation coverage rate targets for DTP3, Hib3 and measles vaccines achieved by the WHO regions of Europe, the Americas and South-East Asia.

High immunisation rates across many LMICs are likely due to the long-standing Expanded Program on Immunization and coordinated efforts from multiple global partners to increase access to essential childhood vaccines [27]. Despite this, substantial numbers of children remain unvaccinated with over 13 million infants each year receiving no vaccines, the so-called “zero dose” children [28]. The SDGs pledge to “leave no one behind” [29] means that it is important to identify these “zero dose” children, as they are left vulnerable to higher rates of disease and death. Children receiving no vaccinations are also associated with other socioeconomic inequities, namely being from the poorest households, living in remote areas or conflict settings, and with mothers who receive no formal education or antenatal care [28,30]. Additional metrics within the GAPPD indicators, such as the percentage of children who have received zero vaccinations, will help identify these children and allow for targeted interventions. Measles cases and outbreaks can be further used as tracers to identify weaknesses in immunisation programs and subpopulations at higher risk of malnutrition and poor access to services [28]. Addressing the issues that underpin the immunisation inequities however, will require cross-sector collaboration, integrated with primary health care services to deliver preventive health care including immunisation at every point of contact.

EBF data reviewed were mainly from African countries. However, findings were similar to a recent Lancet series that showed EBF rates in LMICs at 37% [31]. Barriers to EBF in LMICs are multi-faceted with influencing factors at individual, community and societal levels [32,33]. Evolving maternal and neonatal health factors, increasing numbers of women in the workforce, inadequate health systems and support for breastfeeding, cultural attitudes, and marketing for breastmilk substitutes are some of the factors at play [33]. Unpacking barriers to breastfeeding in the local context, may help countries better plan their policies and programs with combined interventions addressing multiple levels of maternal influence shown to have larger, positive effects on breastfeeding outcomes [32].

Access to an appropriate health care provider was overall higher than rates of antibiotic treatment. Appropriate antibiotic treatment requires clinical judgement from a trained health care provider. The majority of data were from caregiver reports based on symptoms. The lack of specificity and the potential of recall bias makes it difficult to determine the true prevalence of pneumonia and appropriate treatment in the community. A study from Vietnam demonstrated that many children presenting to hospital with upper respiratory symptoms were misclassified as pneumonia and received unnecessary antibiotic treatment prior to hospital presentation [34]. Additional data from health facilities may provide more accurate data on pneumonia case-management practices.

The most updated resources found for GAPPD indicator monitoring were the annual IVAC/JHBSPH reports. Their calculated “GAPPD scores” from combined existing national data sources allow for comparison of GAPPD coverage rates between countries with analyses of time trends, to display progress towards the 2025 coverage targets. Pooling data sources may enhance the richness of information collected, but it can also lend to result bias and inaccurate assumptions. In this case, most data points needed to calculate annual national GAPPD scores were not collected each year, and hence may not have truly reflected the progress in GAPPD indicator coverage for that year. Countries with missing data had the relevant indicator either excluded or counted as 0% coverage for the GAPPD score calculation, potentially leading to skewed results. A review of GAPPD indicators with guidance and clarity on monitoring mechanisms may help countries with more sustainable data collection and reporting activities.

GAPPD indicators to date, have rarely been reported at the subnational level. Reporting at the national level omits the variability in coverage rates that occurs within a country, hiding pockets of inequity and high-risk subpopulations. Iuliano et al. was the only study to compare subnational data, demonstrating very disparate results between Nigerian states, Lagos and Jigawa [24]. This difference is likely in part, a result of inequitable access to health care and standards of living. Lagos is a relatively small urban area with three tertiary hospitals and 85.4% of its population in the highest wealth quintile; whereas Jigawa is a rural region spanning 22 410 km^2^ with only one tertiary hospital and 50.3% of its population in the lowest wealth quintile [35]. Expanded reporting of subnational level data would assist in identifying high risk subpopulations such as Jigawa and reveal underlying factors for inequity, to guide targeted interventions and policy changes.

Our review found that the DHS and MICS were commonly used data sources for reporting GAPPD indicators. Their strengths lie in the longevity, extensive reach and standardised methods used to collect nationally representative data [36,37]. Although they were used mainly for national-level reporting, data from these surveys have the capacity to present progress of equity gaps in GAPPD indicators amongst subgroups within countries. Previous studies from Vietnam and Nepal using data from the DHS and MICS between 2000 to 2014, found national level improvements in immunisation coverage and health seeking behaviours for acute illnesses [38,39]. However, when the data was disaggregated by household socioeconomic status, geographical location, parental education and ethnicity, inequities amongst these subpopulations persisted over the 13 to 14-year period. However, there should be caution in using household surveys in isolation to monitor GAPPD indicators. Although the DHS and MICS adhere to scientifically sound sampling methods, they rely on fixed dwellings, and hence exclude the highly vulnerable groups without formal addresses. Household surveys also rely heavily on caregiver recollection leading to recall bias for GAPPD indicators such as case management for pneumonia. Additional studies focused on these hard-to-reach populations and using data from health facilities where available, may be valuable in mapping GAPPD indicators and pneumonia outcomes in these marginalised communities.

It is timely for a review of the GAPPD-pneumonia targets and their indicators which were set a decade ago. The current GAPPD indicators mainly direct interventions at the community level. Severe pneumonia however, requires access to and treatment in hospital. Although hypoxaemia is a strong predictor of mortality for children presenting to hospital with pneumonia, oxygen was only listed by the WHO as an essential medicine for treatment of hypoxaemia as recently as 2017 [40-43]. In many LMICs, access to supplementary oxygen is low. Barriers to appropriate use of available oxygen supply such as lack of staff training and oxygen saturation monitoring further preclude optimal utilisation [44]. A future GAPPD target should include universal access to uninterrupted oxygen supply for severe pneumonia, where “access” encompasses availability, affordability and appropriate use [45]. Indicators to monitor progress of oxygen access in health facilities will highlight the vital roles pulse oximetry and oxygen therapy play in treating pneumonia and should contribute to the goal of reducing pneumonia related deaths.

Prevalence of malnutrition (undernutrition, overweight/obesity, micronutrient deficiencies) are SDG indicators in the overall SDG 2 to end hunger by 2030 [29]. Undernutrition (underweight, stunting and wasting) is a major risk factor for pneumonia and pneumonia-related mortality but is not a current GAPPD indicator [46-50]. Decreasing childhood undernutrition would contribute to SDG 3.2 of ending preventable child deaths along with SDG target 2.2 of ending all forms of malnutrition [29]. The recent 2020 Global Nutrition Report showed that progress towards global nutrition targets were, however, not on course with 149 million children under five stunted and 49.5 million children wasted at the end of 2018 [51]. Interventions targeting undernutrition require multisectoral partnerships, addressing causes of undernutrition across the life-course [52]. Monitoring progress with measurable indicators is critical to inform interventions and assess impact. Whilst the current GAPPD indicator of complementary feeding may act as a proxy for undernutrition, it is subjective and difficult to collect information on. Undernutrition, as defined through anthropometric measures, are objective, performed routinely within existing programs and more readily reflect severe and chronic nutritional deprivation [53] and hence can serve as valuable and feasible GAPPD indicators.

### Limitations

There were several limitations in this review. Our systematic review only included peer-reviewed publications from 2015 onwards, resulting in limited interpretation of time trends. The WHO GAPPD monitoring database and IVAC/JHBSPH reports had data extracted from 2010/2011 onwards, allowing for more analysis of trends in coverage rates over time but was still limited by lack of data availability for most GAPPD indicators. We excluded single site studies, as it was thought the methods of data collection and results would not have been representative of the population. This may have resulted in the inadvertent omission of studies on vulnerable subgroups.

## CONCLUSIONS

Pneumonia specific GAPPD indicator progress is not widely published in the current literature. Progress towards the GAPPD targets as measured by the GAPPD-pneumonia indicators over the last five to 10 years has been slow. There is a lack of subnational level reporting and analysis of data, required to determine levels of inequity and identify at-risk populations. The GAPPD indicators, along with recommended interventions, have not been revised since 2013. Oxygen access and undernutrition, both with substantial impacts on pneumonia outcomes, are notably absent. The addition of these indicators along with subnational data analysis would enhance future GAPPD monitoring programs towards achieving the overall goals of ending preventable childhood deaths.

## Additional material


Online Supplementary Document
