# Supplementary material for: Slow progress towards pneumonia control for children in low-and-middle income countries as measured by pneumonia indicators: A systematic review of the literature
Source: J Glob Health. 2022 Oct 25;12:10006. doi: 10.7189/jogh.12.10006 (PMC9595578; doi:10.7189/jogh.12.10006)
Supplement: Online Supplementary Document [file jogh-12-10006-s001.pdf]

Supplementary appendix

Appendix S1 – MEDLINE search strategy for systematic review on progress in pneumonia control for children in LMICs: countries reporting on GAPPD indicators

MEDLINE (Ovid) Search strategy

#1	(gappd or (Global-action-plan adj7 pneumonia)).tw,kf.
#2	exp *Pneumonia/
#3	((lower-respiratory adj3 infection*) or pneumonia or pneumonias or lung-inflammation* or lobitis or nonspecific-inflammatory-lung-disease* or peripneumonia or pleuropneumonia or pleuropneumonitis or pneumonic-lung* or pneumonic-pleurisy or pneumonic-pleuritis or pneumonitides or pneumonitis or pulmonal-inflammation* or pulmonary-inflammation* or pulmonic-inflammation* or bronchiolitis).tw,kf.
#4	*Pneumococcal Infections/
#5	2 or 3 or 4
#6	*Global Health/
#7	exp *"Delivery of Health Care, Integrated"/
#8	*International Cooperation/
#9	*consensus development conferences as topic/
#10	(national* or global* or coordinated or co-ordinated or coordination or co-ordination or coalition or integrated or international*).tw,kf.
#11	6 or 7 or 8 or 9 or 10
#12	(intervention* or action* or plan* or goal* or strateg* or program* or policy or policies or guideline* or consensus).tw,kf.
#13	*evaluation studies as topic/ or *program evaluation/
#14	exp *immunization/ or *Immunization Programs/
#15	(hiv and (anti-retroviral* or antiretroviral*) and pregnan*).tw,kf,hw
#16	exp *Anti-Bacterial Agents/tu
#17	*Breast Feeding/
#18	*Breast Feeding/
#19	*Health Knowledge, Attitudes, Practice/ or *"treatment adherence and compliance"/or exp *"patient acceptance of health care"/
#20	(antibiotic* or amoxicillin* or amoxycillin* or gentamicin* or gentamycin* or cotrimoxazole or cotrimoxazole or breastfeeding or breast-feeding or air-pollution or (clean adj3 fuel*) or care-seeking).tw,kf.
#21	(pnu-im?une or pnuim?une or pcv10 or pcv-10 or pcv13 or pcv-13 or prevenar13 or prevenar-13 or prevnar13 or prevnar-13).tw,kf.
#22	((10-valent or ten-valent or 13-valent or thirteen-valent) and (pneumococcal adj5 vaccine*)).tw,kf.
#23	((Hib or Haemophilus-influenzae-type-b or DTP3 or Diphtheria-tetanus-pertussis or measles) and (vaccine* or immuni#ation*)).tw,kf.
#24	exp *Pneumococcal Vaccines/ or *Haemophilus Vaccines/ or exp *Measles Vaccine/ or *Diphtheria-Tetanus-Pertussis Vaccine/
#25	12 or 13 or 14 or 15 or 16 or 17 or 18 or 19 or 20 or 21 or 22 or 23 or 24
#26	(mo or pc).fs.
#27	exp *mortality/ or *Death/ or *infant death/
#28	(mortalit* or death* or surviv* or prevent* or control*).tw,kf.
#29	*preventive medicine/
#30	26 or 27 or 28 or 29
#31	*united nations/ or exp *world health organization/
#32	(UN or WHO or United-Nation* or World-Health-Organization*).tw,kf.
#33	developing countries/
#34	(austere or (limited adj2 resource*) or (low adj2 resource*) or (transitioning adj econom*) or (third adj world) or LMIC or LMICs or (lami adj countr*) or (transitional adj countr*) or (low adj gdp) or (low adj gnp) or (low adj gross adj domestic) or (low adj gross adj national) or ((emerging or

	developing or (low adj income) or (middle adj income) or (low adj3 middle) or underdeveloped or under-developed or (less* adj developed) or underserved or under-served or deprived or poor*) and (countr* or nation*1 or econom* or population or world))).tw,kf.
#35	exp africa/
#36	americas/ or exp caribbean region/ or exp central america/ or latin america/ or mexico/ or exp south america/
#37	europe/ or exp europe, eastern/ or exp transcaucasia/
#38	antarctic regions/ or exp atlantic islands/ or exp indian ocean islands/ or exp pacific islands/
#39	New Guinea/
#40	asia/ or exp asia, central/ or asia, southeastern/ or borneo/ or cambodia/ or east timor/ or indonesia/ or laos/ or malaysia/ or mekong valley/ or myanmar/ or philippines/ or thailand/ or vietnam/ or asia, western/ or bangladesh/ or bhutan/ or india/ or middle east/ or afghanistan/ or iran/ or iraq/ or jordan/ or lebanon/ or oman/ or saudi arabia/ or syria/ or turkey/ or yemen/ or nepal/ or pakistan/ or sri lanka/ or far east/ or china/ or tibet/ or exp korea/ or mongolia/
#41	(Afghanistan or Albania or Algeria or Angola or Antigua or Argentina or Armenia* or Aruba or Azerbaijan or Bahrain or Bangladesh or Barbados or Barbuda or Belarus or Byelarus* or Byelorussian or Belorussian or Belorus* or Belize or Benin or Bhutan or Bolivia or Bosnia or Botswana or Brasil or Brazil or Bulgaria or (Burkina adj Fas*) or (Upper adj Volta) or Burma or Burundi or Cambodia or Khmer or Kampuchea or Cameron* or Cameroon* or (Cape adj Verde) or (Cabo adj Verde) or (Central adj African adj Republic) or Chad or Chile or China or Colombia or Comoros or (Comoro adj Island*) or Comores or Mayotte or Congo or Kongo or (Cook adj Island*) or (Costa adj Rica) or (Cote adj D'ivoire) or Croatia or Cuba or Cyprus or Czech* or Djibouti or Dominica or Dominican or (East adj Timor) or (East adj Timur) or Ecuador or Egypt or El-Salvador or (Equatorial adj Guinea) or Eritrea or Estonia or Ethiopia or Fiji or (French adj Somaliland) or Futuna or Gabon or (Gabonese adj Republic) or Gambia or Gaza or (Georgia* adj Republic) or Ghana or Grenada or Guam or Guatemala or Guinea or Guiana or Guyana or Haiti or Herzeg* or Hercegovina or Honduras or Hungary or India or Indonesia or Iran or Iraq or (Ivory adj Coast) or Jamaica or Jordan or Kazakh* or Kenya or Kiribati or Korea or Kosovo or (Kyrgyz adj Republic) or Kyrgyzstan or Kirghizia or Kirghiz or Kirgizstan or Laos or (Lao* adj2 Democratic adj Republic) or (Lao* adj PDR) or Latvia or Lebanon or Lesotho or Basutoland or Liberia or Libya or Lithuania or Macedonia or Madagascar or (Magalasy adj Republic) or Malawi or Malay* or Sabah or Sarawak or Maldives or Mali or (Marshall adj Island*) or Mauritania or Mauritius or (Agalega adj Island*) or Mexico or Micronesia or Moldov* or Mongolia or Montserrat or Montenegro or Morocco or Ifni or Mozambique or Myanma* or Namibia or Nauru or Nepal or (Netherlands adj Antilles) or (Dutch adj Antilles) or (New adj Guinea) or (New adj Caledonia) or Nicaragua or Niue or Niger or Nigeria or (Northern adj Mariana adj Island*) or Nyasaland or Oman or Pakistan or Palau or Panama or (Papua adj New adj Guinea) or PNG or Palestine or Paraguay or Peru or Philipines or Philippines or Phillipines or Phillippines or Poland or (Puerto adj Rico) or Yemen or Romania or Roumania or Rumania or Russia* or Rwanda or Ruanda or (Saint adj Kitts) or (St adj Kitts) or Nevis or (Saint adj Vincent) or (St adj Vincent) or Grenadines or Samoa* or (Navigator adj Island*) or (Saint adj Lucia) or (St adj Lucia) or (Saint adj Helena) or (St adj Helena) or (Sao adj Tome) or (Saudi adj Arabia) or Senegal or Serbia or Seychelles or (Sierra adj Leone) or Slovenia or Slovak* or (South adj Africa) or (Solomon adj Island*) or Somalia or (Sri adj Lanka) or Ceylon or Sudan or Surinam* or Swaziland or Syria or Tajikistan or Tadzhikistan or Tadjikistan or Tadzhik or Tanzania or Thailand or Tibet or Timor-Leste or Togo or (Togolese adj Republic) or Tokelau or Tonga or Trinidad or Tobago or Tunisia or Turkey or Turkmenistan or Turkmen or Tuvalu or Uganda or Ukraine or Uruguay or Urundi or USSR or (Soviet adj Union) or "Union of Soviet Socialist Republics" or Uzbekistan or Vanuatu or (New adj Hebrides) or Venezuela or Vietnam or (Viet adj Nam) or (Wallis adj2 Futuna) or (United adj Arab adj Republic) or (West adj Bank) or (West adj Indies) or Yemen or Yugoslavia or Zaire or Zambia or Zimbabwe or Rhodesia).tw,kf.
#42	(africa or americas or caribbean or (central adj America) or (latin adj America) or (south adj America) or (eastern adj Europe) or Transcaucasia or antarctic or (atlantic adj island*) or (indian adj ocean adj island*) or (pacific adj island*) or polynesia or (central adj asia) or (southeast* adj asia) or (south-east* adj asia) or borneo or mekong or (western adj asia) or (middle adj east) or (far adj east)).tw,kf.

#43	31 or 32 or 33 or 34 or 35 or 36 or 37 or 38 or 39 or 40 or 41 or 42
#44	(newborn* or new-born* or baby or babies or neonat* or neo-nat* or infan* or toddler* or pre-schooler* or preschooler* or kinder or kinders or kindergarten* or kinder-aged or boy or boys or girl or girls or child or children or childhood or pediatric* or paediatric* or adolescen* or youth or youths or teen or teens or teenage* or school-age* or schoolage* or schoolchild* or schoolgirl* or schoolboy*).af.
#45	5 and 11 and 25 and 30 and 43 and 44
#46	1 or 45
#47	limit 46 to (english language and yr="2015 -Current")

Appendix S2: List of include countries in the WHO GAPPD monitoring database

**WHO Region**	**Country**
African Region (AFR)	Algeria<br>Angola<br>Benin<br>Botswana<br>Burkina Faso<br>Burundi<br>Cabo Verde<br>Cameroon<br>Central African Republic<br>Chad<br>Comoros<br>Congo<br>Côte d'Ivoire<br>Democratic Republic of the Congo<br>Equatorial Guinea<br>Eritrea<br>Ethiopia<br>Gabon<br>Gambia<br>Ghana<br>Guinea<br>Guinea-Bissau<br>Kenya<br>Lesotho<br>Liberia<br>Madagascar<br>Malawi<br>Mali<br>Mauritania<br>Mauritius<br>Mozambique<br>Namibia<br>Niger<br>Nigeria<br>Rwanda<br>Sao Tome and Principe<br>Senegal<br>Sierra Leone<br>South Africa<br>South Sudan<br>Swaziland

	Togo<br>Uganda<br>United Republic of Tanzania<br>Zambia<br>Zimbabwe
Eastern Mediterranean Region (EMR)	Afghanistan<br>Djibouti<br>Egypt<br>Iran (Islamic Republic of)<br>Iraq<br>Jordan<br>Lebanon<br>Libya<br>Morocco<br>Pakistan<br>Somalia<br>Sudan<br>Syrian Arab Republic<br>Tunisia<br>Yemen
European Region (EUR)	Albania<br>Armenia<br>Azerbaijan<br>Belarus<br>Bosnia and Herzegovina<br>Bulgaria<br>Croatia<br>Georgia<br>Kazakhstan<br>Kyrgyzstan<br>Montenegro<br>Republic of Moldova<br>Romania<br>Russian Federation<br>Serbia<br>Tajikistan<br>The former Yugoslav Republic of Macedonia<br>Turkey<br>Turkmenistan<br>Ukraine<br>Uzbekistan
Region for the Americas (AMR)	Argentina<br>Belize<br>Bolivia (Plurinational State of)<br>Brazil<br>Colombia<br>Costa Rica<br>Cuba<br>Dominica<br>Dominican Republic<br>Ecuador<br>El Salvador<br>Grenada<br>Guatemala<br>Guyana<br>Haiti

	Honduras<br>Jamaica<br>Mexico<br>Nicaragua<br>Panama<br>Paraguay<br>Peru<br>Saint Lucia<br>Saint Vincent and the Grenadines<br>Suriname<br>Venezuela (Bolivarian Republic of)
South-East Asia Region (SEAR)	Bangladesh<br>Bhutan<br>Democratic People's Republic of Korea<br>India<br>Indonesia<br>Maldives<br>Myanmar<br>Nepal<br>Sri Lanka<br>Thailand<br>Timor-Leste
Western Pacific Region (WPR)	Cambodia<br>China<br>Cook Islands<br>Fiji<br>Kiribati<br>Lao People's Democratic Republic<br>Malaysia<br>Marshall Islands<br>Micronesia (Federated States of)<br>Mongolia<br>Nauru<br>Niue<br>Papua New Guinea<br>Philippines<br>Samoa<br>Solomon Islands<br>Tonga<br>Tuvalu<br>Vanuatu<br>Viet Nam
